# Secondary Vesical Calculi in a Young Male With Post‐Gunshot Neurogenic Bladder and Prior Colostomy: A Case Report

**DOI:** 10.1002/ccr3.72902

**Published:** 2026-06-12

**Authors:** Syeda Hani Ammad, Govinda Meheshwari, Waleed Ahmad, Abdullah Akram, Mohammed Hammad Jaber Amin

**Affiliations:** ^1^ Karachi Medical and Dental College Karachi Pakistan; ^2^ Jinnah Post Graduate Medical Centre Karachi Pakistan; ^3^ Lahore Medical and Dental College Lahore Pakistan; ^4^ Nawaz Sharif Medical College Gujrat Pakistan; ^5^ Alzaiem Alazhari University Khartoum Sudan

**Keywords:** case report, colostomy, neurogenic bladder, post‐gunshot, secondary vesical calculi, surgery

## Abstract

Bladder stones are highly prone to develop in patients with neurogenic bladder and long‐term indwelling catheterization. Close observation, careful catheter management, and timely intervention are crucial in avoiding complications and recurrence.

## 1 Introduction

Bladder stones make 5% of urinary tract stones. As a rule, they typically occur in the presence of bladder neck obstruction, neurogenic bladder, and foreign object‐related urinary tract infections [1]. Patients with neurogenic lower urinary tract dysfunction are at high risk of recurrent bladder calculi and require close observation [2]. One of the most often used instruments of bladder management in individuals with high‐level spinal cord injury who cannot intermittently catheterize their bladder is through indwelling catheters. The purpose of the indwelling catheters is to prevent bladder overdistension, which might initiate autonomic dysreflexia in people with injuries at or above T6. Indwelling catheters are unfortunately liable to encrustment and result in the development of bladder stones [3]. It is an uncommon case of secondary bladder calculi in a young patient with post‐traumatic neurogenic bladder and prior colostomy.

Although bladder stones are frequent in elderly men with bladder outlet obstruction, they are rare in young patients with traumatic neurogenic bladder. This case is uncommon because of the young age, the presence of a post‐traumatic neurogenic bladder, and the fact that the patient had undergone abdominal surgery before and had 14 stones, which is of great relevance as it can be used in surgical management and preventive interventions.

## 2 Case History/Examination

A 19‐year‐old man presented to the urology clinic complaining of weakness in the lower limbs and urinary incontinence. He stated that these problems had occurred since he sustained a gunshot wound to the lower back 1.5 years ago. The bullet also resulted in spinal trauma leaving him with neurogenic bladder dysfunction and permanently dependent upon an indwelling Foley catheter to drain his bladder. After the trauma, he had a laparotomy and colostomy formation, which was still functioning. His daily needs, such as catheter care, were managed by his family, and he claimed he was compliant with routine catheter changes in the past, but still occasional bouts of urinary tract infection had been experienced. He has no smoking or alcohol history or recreational drug use, and his general nutritional status was overall satisfactory.

Physical examination revealed a patient who was oriented and alert and had stable vital signs. Examination of the abdomen showed a midline laparotomy scar and colostomy in the left lower quadrant. No evidence of distension, tenderness, or organomegaly was observed. Neurological examination revealed the presence of spasticity in both lower extremities with limited motor strength (Grade 3/5) and the loss of reflexes indicative of long‐standing spinal cord injury. The level of injury was below the level of sensory examination. A Genitourinary examination revealed the presence of an indwelling Foley catheter that was patent and draining clear urine.

## 3 Investigations/Differential Diagnosis/Treatment

Laboratory tests also involved urinalysis which revealed a high alkaline pH (7.0), high specific gravity (1.030), proteinuria (++) and a large number of pus cells indicating a possible chronic colonization in the urinary tract. The complete blood count, renal function tests, and serum electrolytes were normal (Table 1). Kidney, ureters, and bladder ultrasonography showed several vesical calculi, the largest 2.1 cm, mild thickening of the bladder wall, and minimal ascites. Both kidneys were found to be normal in size and echotexture without hydronephrosis. Such results were in accordance with secondary calculi of the bladder that are related to the chronic presence of catheters in the bladder in the context of neurogenic bladder.

**TABLE 1:** Relevant laboratory findings of the patient on admission.

Urine examination	Result	Normal range
Color	Yellow	—
Reaction pH	7.0	5.0–6.0
Specific gravity	1.030	1.010–1.025
Protein	++	Negative
Glucose	Nil	Nil
Ketone bodies	Nil	Nil
Urobilinogen	Normal	Normal
Bilirubin	Nil	Nil
RBCs	2–4/HPF	0–2/HPF
Pus cells	Numerous/HPF	0–5/HPF
Epithelial cells	Occasional/HPF	Few
Casts	Nil	Nil

Abbreviation: HPF = high power field.

The differential diagnosis involved calculi caused by infection, foreign body deposition on the Foley catheter, and metabolic stones. Chronic urinary bacterial colonization and alkaline urine were regarded as the key contributing factors in the formation of stones.

**FIGURE 1:**
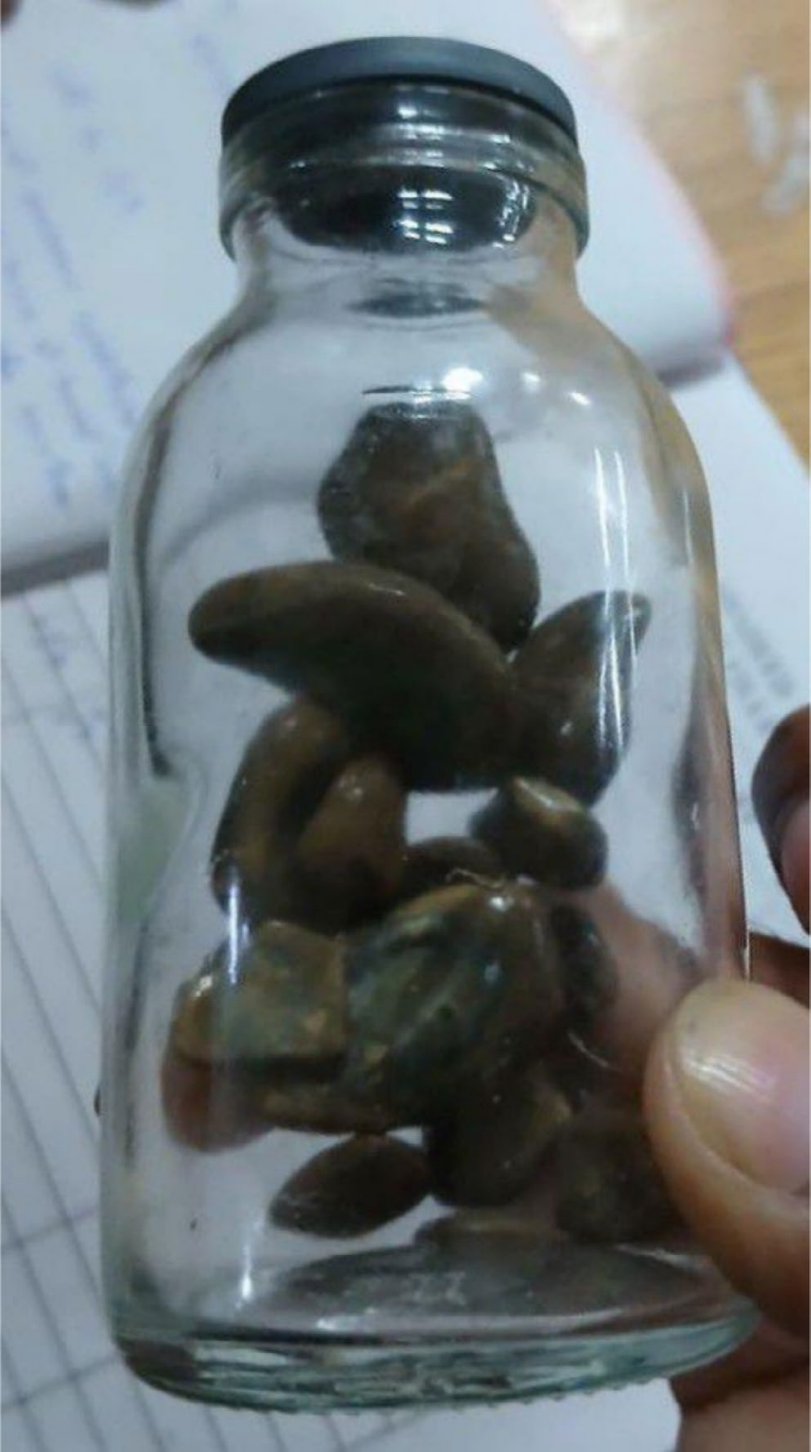
Fourteen vesical calculi retrieved following percutaneous cystolithotomy in a young male with post‐gunshot neurogenic bladder.

Upon counseling and preoperative preparation, the patient went through percutaneous cystolithotomy under general anesthesia. The operation team used a suprapubic incision of 2 cm and took great care to separate the subcutaneous tissue and rectus muscle to reach the bladder. Fourteen calculi were totally removed (Figure 1), and the bladder was stitched up in layers. A Foley catheter was reinserted and aseptic precautions were closely followed. The patient did not have any intraoperative complications, and he tolerated the procedure well. Analgesia, urine output monitoring, and catheter hygiene were incorporated as postoperative care. He was sent home in good health, but with the recommendation of frequent change of catheter and follow up imaging to check on recurrence.

## 4 Conclusion and Results

The surgery was uneventful, and postoperative recovery was smooth. The patient was advised to continue regular catheter care and follow‐up after being discharged in stable condition. The patient was still asymptomatic at 3‐month follow‐up, and the ultrasonography revealed no new stones. Frequent catheter replacement and aseptic catheter care were underlined, and the role of prevention measures in the treatment of neurogenic bladder patients was discussed.

Young patients with post‐traumatic neurogenic bladder having numerous secondary bladder stones is extremely uncommon. This case illustrates the fact that percutaneous cystolithotomy is a safe and effective method of management even in people who have had previous abdominal surgery. Such preventive measures as frequent catheter maintenance, early imaging, and aseptic technique are essential to minimize the recurrence risk and related complications.

## 5 Discussion

Only 5%–10% of all urinary tract stones are caused by bladder calculi, which are uncommon in young people without any underlying anatomical or functional abnormalities. They are usually caused by infection, stasis of urine, or by the presence of foreign bodies. The incomplete emptying and chronic urine retention are identified as risk factors that cause neurogenic bladder following spinal trauma. Urinary stasis and frequent infections in neurogenic bladder facilitate alkaline urine and bacterial biofilm formation, which promotes crystallization and subsequent stone formation. Mohr et al. endorse this mechanism when they reported that neurogenic lower urinary tract dysfunction is a significant risk factor for bladder stones [2].

Even long‐term indwelling catheterization further increases the risk of bacterial colonization, encrustation, and consequent development of stones. Werneburg suggests that the duration of catheterization is the most significant element in the progression of bacteriuria, with the chances of it being 37% each day [4]. The constant presence of bacterial biofilm and deposition of salts give the nidus in which stone grows. The process can also be hastened by violations of aseptic catheter technique; Joshi et al. found that failure of aseptic technique can introduce foreign material into the bladder, which encourages encrustment [5]. An overview of the literature indicates that multiple cases of bladder stones in young patients with post‐traumatic neurogenic bladder are extremely uncommon [6]. The majority of cases reported are in middle‐aged or elderly patients with congenital or degenerative neurogenic dysfunction.

The combination of multiple unusual factors makes this case particularly impressive. The young age of the patient (19 years old) is also unusual since the stones in the bladder are characteristic of middle‐aged or older men. The underlying etiology, a neurogenic bladder, as a result of a gunshot, is itself unusual, when compared to the causes of neurogenic dysfunction that are congenital or degenerative. The surgery was also complicated by a prior laparotomy and colostomy as well as the existence of 14 vesical calculi. In the eyes of the management, percutaneous cystolithotomy is a good alternative to open cystolithotomy, which is minimally invasive. Percutaneous cystolithotomy was chosen as a minimally invasive and efficient procedure to clear all the stones, especially when several calculi are present. Chatterjee et al. established the fact that percutaneous cystolithotomy is an established procedure in the retrieval of bladder stones in adults [7], and Metwally et al. reported that percutaneous cystolithotomy has proven to be a well‐established procedure with high effectiveness, especially in bladder stones of great size or multiple ones [8]. Our patient had all the 14 calculi removed with minimum postoperative complications and quick recovery.

Prevention is also vital in patients with neurogenic bladder other than management. Another key point to note in this case is the early imaging in a young patient with a history of catheterization, the use of a high level of aseptic technique during catheter handling, and timely surgical removal of multiple stones. One of the limitations of this report is that there was no advanced imaging, including CT scans, to further characterize the stone burden and the urinary tract anatomy; however, ultrasonography was the primary imaging modality and it was adequate to make the diagnosis in the clinical setting. Longer follow‐up studies are required in future to determine the risk of recurrence and the long‐term outcomes of similar patients. The prevention measures in these patients should aim at routine replacement of catheters, strict aseptic practice, surveillance imaging and early treatment of urinary tract infections to minimize the risk of recurrence.

## Author Contributions

**Syeda Hani Ammad:** conceptualization, writing – original draft. **Govinda Meheshwari:** supervision, writing – review and editing. **Waleed Ahmad:** methodology, writing – original draft, writing – review and editing. **Abdullah Akram:** resources, writing – original draft. **Mohammed Hammad Jaber Amin:** investigation, project administration, writing – review and editing.

## Funding

The authors have nothing to report.

## Disclosure

Declaration of AI Content: This case study was not generated by AI tools. While AI was utilized to enhance the professionalism and readability of the content, it was not used extensively to the extent that the work appears AI‐generated. The primary content, analysis, and conclusions are the result of the author's original work.

## Consent

The patient provided written consent to allow researchers to use their anonymously processed information in this article.

## Conflicts of Interest

The authors declare no conflicts of interest.

## Data Availability

Within the article, the data used to support the findings of the present study are available. Any additional information is confidential and cannot be disclosed to the audience.
